# Characterisation and evaluation of pharmaceutical solvates of Atorvastatin calcium by thermoanalytical and spectroscopic studies

**DOI:** 10.1186/1752-153X-6-114

**Published:** 2012-10-06

**Authors:** Renu Chadha, Astha Kuhad, Poonam Arora, Shyam Kishor

**Affiliations:** 1 University Institute of Pharmaceutical Sciences, Panjab University, Chandigarh, 160014, India; 2 Department of Chemistry, Janta Vedic (PG) College, Baraut, Meerut, Uttar Pradesh, India

**Keywords:** Solvate, Recrystalliztion, Heat capacity, Calorimetry, ^13^C /MAS solid state NMR

## Abstract

**Background:**

Atorvastatin calcium (ATC), an anti-lipid biopharmaceutical class II drug, is widely prescribed as a cholesterol-lowering agent and is presently the world’s best-selling medicine. A large number of crystalline forms of ATC have been published in patents. A variety of solid forms may give rise to different physical properties. Therefore, the discovery of new forms of this unusual molecule, ATC, may still provide an opportunity for further improvement of advantageous properties.

**Results:**

In the present work, eight new solvates (Solvate I-VIII) have been discovered by recrystallization method. Thermal behaviour of ATC and its solvates studied by DSC and TGA indicate similar pattern suggesting similar mode of entrapment of solvent molecules. The type of solvent present in the crystal lattice of the solvates is identified by GC-MS analysis and the stoichiometric ratio of the solvents is confirmed by ^1^HNMR. The high positive value of binding energy determined from thermochemical parameters indicates deep inclusion of the solvent molecules into the host cavity. The XRPD patterns point towards the differences in their crystallanity, however, after desolvation solvate II, III, IV, V and VIII transform to isostructral amorphous desolvated solvates. The order of crystallinity was confirmed by solution calorimetric technique as the enthalpy of solution is an indirect measure of lattice energy. All the solvates behaved endothermically following the order solvate-VIII (1-butanol solvate) < solvate-I (isoproplyate) < solvate-V (methanol solvate) < solvate-III (ethonalate) < solvate-VI (acetone ethanol solvate) < solvate-IV (t-butanol solvate) < solvate-II (THF solvate) < solvate-VII (mixed hemi-ethanol hydrate). The positive value of the heat capacity of the solvate formation provides information about the state of solvent molecules in the host lattice. The solvents molecules incorporated in the crystal lattice induced local chemical environment changes in the drug molecules which are observed in ^13^CP/MAS NMR spectral changes.

**Conclusions:**

Aqueous solubility of solvate-VIII was found to be maximum, however, solvate-I and VIII showed better reduction in total cholesterol and triglyceride levels as compared to atorvastatin against triton-induced dyslipidemia.

## Background

Active pharmaceutical ingredients as well as excipients may exist in different solid forms which is of great importance for pharmaceutical development [1]. One important type of solid forms are solvates. Over the past decade, many different solvates of pharmaceutical materials with readily discernible physical and chemical characteristics, and marked differences in their pharmaceutical performance have appeared. Solvates are defined as crystalline multi-component system in which a solvent(s) is accommodated by the crystal structure in stoichiometric and non-stoichiometric manner [2]. The formation of organic solvates instead of hydrates plays a significant role since the pharmaceutical substances are often exposed to organic solvent during production and processing. In addition, the transformation behaviour of solvates during crystallization and storage is often complicated and difficult to understand completely. The relative stability of the solvated and non-solvated forms depends upon the temperature, pressure and thermodynamic activity of the solvent. Therefore, a study of these considerations is important in order to design or control a process that yields a solid form with the desired properties. The isostructural unsolvated form generated by desolvation is called isomorphic desolvate or desolvated solvate [2]. Hence, the screening for various crystal modifications has been increasingly emphasized in pharmaceutical industry as it provides valuable knowledge necessary for further development of the drug formulation [3-5].

**Figure 1:**
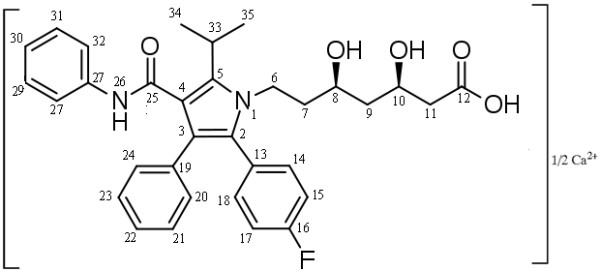
Molecular structure of ATC molecule, showing the numbering of the carbons and nitrogen.

Atorvastatin, 3-hydroxy-3-methyl-glutaryl-coenzyme A (HMG-CoA) reductase inhibitor (Figure 1), a cholesterol-lowering agent for the treatment of hypercholesterolemia is a synthetic statin, which catalyzes the rate-limiting step in cholesterol biosynthesis. Atorvastatin is marketed as its calcium salt in crystalline trihydrate. However, the sky soaring demand of ATC and an interesting structure of this drug directed the pharmaceutical scientist towards a high number of patents and many publications offering different crystalline forms as well as processes to generate alternative forms [6]*.* A variety of solid forms may give rise to different physical properties. The successful utilization of a crystal form of significantly greater thermodynamic activity (i.e., solubility) than the stable modification may provide, in some instances, therapeutic blood levels from otherwise inactive drugs [7-10]. However, ATC, belongs to an anti-lipid class II drug, not more emphasis have been laid to determine the solubility of the new form generated. Besides these none of the publications report the in-vivo activity of the new recrystalised forms. Therefore, the discovery of new crystalline solvates of this unusual molecule, ATC, may still provide an opportunity for further improvement of advantageous properties. Thus the present investigation deals with the preparation of different solvates of ATC by solvent recrystallization method and to characterize them using established solid state and solution phase techniques. The solvates are further evaluated by in-vivo studies using Triton induced hyperlipidemia to select the most suitable solvate. The objective was to gain a better understanding of the solid state behaviour of ATC solvates before and after desolvation which might enable the development of a more consistent and efficacious ATC oral formulation.

## Results and discussion

The various forms of ATC were subjected to different techniques for their exact characterization. TGA provides insight about the amount of solvent actually present and DSC provides information on the energetics, of how it interacts within the crystal lattice of parent molecule [11]. As all the solvates have been obtained by recrystallization using more than one solvent and one or two step desolvation has been observed in the TGA, it was necessary to confirm the type of solvent associated with the drug molecule. To confirm this, GCMS analysis has been performed [12]. Initial characterization and stoichiometry of the various solvates have been established by DSC, TGA, GCMS and ^1^H NMR analysis. Existence of solvents were further confirmed by ^13^C /MAS solid state NMR.

### Characterization by Differential Scanning Calorimetry (DSC), Thermogravimetric Analysis (TGA), GCMS and Karl-Fisher analysis

**Figure 2:**
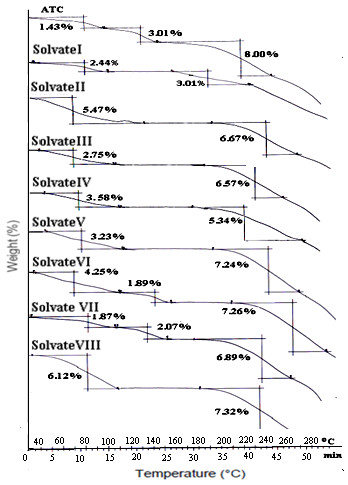
TGA of ATC and its solvates.

**Figure 3:**
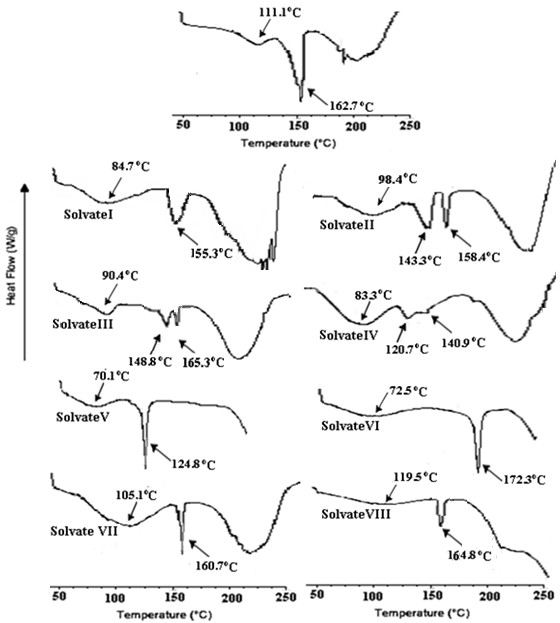
DSC thermograms of ATC and its solvates.

TGA analysis of ATC and its different forms at heating rate of 10°C/min (Figure 2) was studied to elucidate the mass loss associated with the thermal transitions. The commercial sample revealed three steps mass loss in the temperature range 40-100°C, 105-140°C and 170-260°C respectively. The first step temperature range indicates it to be loosely bound water. Subsequently, the second step is associated with loss of water of hydration followed by total mass loss (above 210°C) due to its degradation. Total mass loss of 4.44% for the first two steps confirm it to be a trihydrate and corresponds to ATC-1 as described [13]. The DSC curve of commercial atorvastatin at 10°C/min exhibited a broad endotherm ranging from 70-140°C indicating the loss of water and a sharp endotherm at 162.78°C (Figure 3) which is due to melting of ATC and is supported by TGA. The DSC and TGA of ATC confirm it to be a trihydrate which melts at 162.78°C.

The TGA of solvates I, II, III, IV, V and VIII showed mass loss in two steps. The first step in the range 40-120°C corresponds to desolvation whereas the second dip is associated with decomposition of the solvates. The DSC thermograms of the prepared solvates showed a single broad endothermic peak with slight variation in onset temperatures followed by not so sharp melting endotherms. Mass losses of 2.44% in TGA of solvate-I suggest 0.5 mole of isopropyl alcohol per mole of the host compound. The broad endothermic peak of solvate-I in the temperature range 40-105°C corresponds to desolvation and a second endotherm at 155.3°C with a significantly lower ΔH_m_ of 8.03 J/g indicate melting. Solvate-I is obtained from isopropyl alchocol, acetone and water as anti-solvent is subjected to GCMS analysis to ascertain the type of solvent present in the lattice. A single peak at 2.28 min corresponding to the retention time for isopropyl alcohol is obtained. This suggests that solvate-I contain isopropyl alcohol while acetone is not associated with the crystal lattice. Besides this, incorporation of water from the recrystallization solvent was ruled by Karl-Fisher analysis. This was immediately followed by decomposition. The above analysis reveals it to be isopropyl alcohol solvate.

A mass loss of 5.5% (55-130°C), 2.7% (50-120°C) and 3.6% (50-110°C) has been demonstrated in the TGA plots of solvate II, III and IV respectively. In the DSC thermograms of solvate II, III (obtained from ethanol) and IV two endothermic events have been observed, a flat and broad one at lower temperature followed by somewhat sharper one. The flat and broad endothermic peaks were observed with a maxima at 90(±8)°C due to solvent loss as suggested by TGA. The desolvation is succeeded by a small endothermic peak prior to the melting and is attributed to solid-solid phase transition (molecular reorientation) to a new polymorphic form which melts at 158°C, 165°C and 140°C in solvate II, III and IV respectively as no mass loss has been observed in this range in TGA. In the similar way, in GCMS analysis of solvate-II (obtained from THF) showed a retention time at 2.32 min which corresponds to retention time for THF. Solvate-IV (obtained from t-butanol using toluene as antisolvent) showed major peak at 2.73 min analogous to retention time for t-butanol. The desolvation temperature ranges and the percent mass loss in TGA for these three solvates indicate solvate-II and solvate-III to be THF and ethanol solvates respectively with 1:1 host to guest stoichiometry (Table 1). The solvate-IV is a tert-butanol solvate with 2:1 stoichiometry.

**Table 1:** Thermodynamic parameters obtained from differential scanning calorimetry and thermogravimetric analysis of Atorvastatin calcium and its solvates

	**Desolvation temperature (°C)**	**Melting point (°C)**	**melting Enthalpy (J g**^**-1**^**)**	**Percentage mass loss on desolvation**		**GCMS Retention time (min)**	**Enthalpy of vapourization of pure solvent at 25°C (kJ mol**^**-1**^**) (24)**	**Enthalpy of desolvation per mole of solvent (kJ mol**^**-1**^**)**
				**(experimental from TGA)**	**(calculated)**			
**Atorvastatin (ATC)**	-	162.78	68.05					
**Solvate- I (ATC + 0.5 Isopropyl alcohol)**	84.71	155.3	8.03	2.44	2.57	2.88	44.00	141.41
**Solvate- II (ATC + THF)**	98.89	158.43	8.86	5.47	5.86	2.32	32.47	55.17
**Solvate- III (ATC + Ethanol)**	90.42	165.30	3.775	2.73	2.83	-	38.61	63.34
**Solvate- IV (ATC + 0.5 t-butanol)**	83. 34	140.97	3.58	3.58	3.11	2.73	37.89	136.63
**Solvate- V (ATC + methanol)**	70.15	124.85	36.00	5.85	5.31	-	35.31	54.90
**Solvate-VI (ATC + 1 Acetone +0.5 Ethanol)**	72.53	172.30	32.66	5.66	6.53	1.64	31.30	65.63
						1.57	38.61	
**Solvate-VII (ATC + 0.5 Ethanol + Water)**	105.13	160.70	9.060	3.94	4.37	1.57	38.61	106.61
							40.67	
**Solvate- VIII (ATC + 1- butanol)**	119. 5	164.8	27.16	6.12	6.59	2.80	43.29	61.46

The solvate-V (obtained from methanol) showed broad desolvation endotherm in the temperature range 41.07°C −105.8°C accompanied by mass loss of 3.16% in TGA. Taking into consideration from TGA this form is found to be a methanol solvate with 1: 1 stoichiometry.

A three step mass loss is indicated in solvate VI and VII, where the first two steps correspond to desolvation as these occur around 50-120°C and the third step around 200°C might be due to degradation. The solvate-VI and VII revealed broad desolvation endotherms with maxima at 72.5°C and 105.1°C accompanied by two step mass loss of 4.25%**/**1.89% for solvate-VI and 1.87%**/**2.07% for solvate-VII in the TGA. Solvate-VI (obtained from acetone, THF and ethanol) showed two major peaks eluting at 1.57 min and 1.64 min in the GCMS analysis. It was easily concluded that the solvents associated with solvate-VI were ethanol and acetone [14]. Solvate-VII (obtained from ethanol and water as an antisolvent) showed major peak at 1.57 min which corresponds to retention time for ethanol. As water has been used as anti-solvent the Karl-Fisher(KF) analysis was used to determine the water content if present. KF analysis indicated that the sample contained 1.48% water. The calculations suggest solvate VI to be a 2:2:1 acetone:ethanol solvate, while solvate-VII is a mixed water:ethanol solvate with 2:2:1 stoichiometry.

Solvate-VIII (obtained from mixture of ACN, isopropylalcohol and 1-butanol) showed elution peak at 2.80 min in GCMS analysis corresponding retention time for butanol, confirming the presence of butanol in the crystal. The solvate-VIII show desolvation endotherm at 119.5°C and an accompanying mass loss of 6.12% in TGA. The calculation shows that solvate-VIII is butanol solvate with 1:1 stiochiometry.

### ^1^H NMR analysis

To confirm the identity and proportion of solvent of crystallization, all the solvates were subjected for ^1^H NMR analysis [15]. The ^1^H NMR of ATC showed total signals for 32 protons (^1^H NMR corresponding to each form are provided in the Additional file 1: Figure S1).

The ^1^H NMR of solvate I showed a signal merged with –CH (position 9) proton of ATC at 3.33 and an extra doublet at δ1.10, integrating one and three protons respectively which confirmed the presence of half mole of isopropyl alcohol in solvate I. However, ^1^H NMR of solvate II showed signals for THF at δ3.32 and at δ2.09 integrating a total of eight protons. These peaks can be accounted for one mole of entrapped solvent in the crystal of ATC-THF solvate. Regarding solvate III an extra quartet integrating two protons at δ3.54 and an extra triplet at δ1.10 integrating three protons confirmed presence of one mole of ethanol in solvate. The ^1^ H NMR of solvate IV revealed singlet at δ1.25 integrating nine protons of t-butanol. Both hemiethanol hydrate and Methanol showed broad signals merged with –CH (position 8) proton of ATC at δ3.57 and δ3.33 ppm respectively. These integrate to two and three protons respectively confirming the association of half mole of ethanol and one mole of methanol in solvate VII and solvate V crystals respectively. Similarly ^1^HNMR of solvate VI showed an extra quartet at δ3.37 and an extra singlet at δ2.10 integrating two and six protons respectively. These signals can be accounted for half mole of ethanol and one mole of acetone in solvate. Similarly ^1^ H NMR of butanol showed an extra triplet integrating two protons at δ3.48, broad signal merged with –CH_3_ (position 12) protons of ATC integrating two protons at δ1.45, sextet integrating two protons at δ1.35 and triplet integrating three protons at δ0.90 respectively. These signals confirmed presence of one mole of butanol in solvate VIII crystals. Thus, the stoichiometry established by TGA and GCMS is confirmed by ^1^H NMR analysis. The results are shown in Table 1.

### Determination of binding energy of solvates

The desolvation temperatures account for the stability of solvates as the desolvation temperature in all these forms is much higher than the boiling point of the corresponding solvent. The quantitative estimate of the stability of these solvates is established by determining the binding energy [1,14,16] for the solvents in all the solvates using equation 1 where the percent mass loss measured over the TG desolvation step of each solvate (Δm_S_%) was related to the enthalpy change calculated over the corresponding DSC desolvation endotherm (ΔH_Sexp_).

(1) $$\Delta H_{S}\;=\;\left[\left(\Delta H_{S\exp}\times \;100\right)\;\Delta m_{s}\%\right]\;\times \;M_{s}$$

where ΔH_S_ (J mol^-1^) is the enthalpy of desolvation (or binding energy) per mole of the bound solvent, M_S_ is the molecular mass of the solvent.

The calculated values of binding energies are given in the Table 1 and are compared with their corresponding enthalpy of vaporization. It can be seen that in all the cases the solvent molecules are strongly bound compared to their interactions in the liquid state suggesting the solvent molecules are tightly bound into the host molecule.

### Scanning electron microscopy

**Figure 4:**
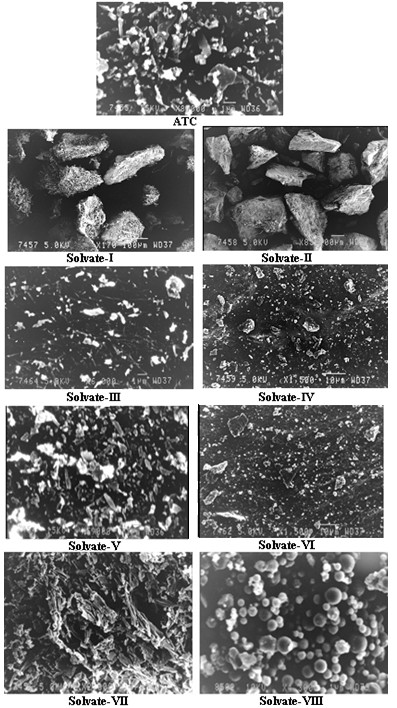
Scanning electron micrographs of ATC and its solvates.

Scanning electron microscopy was used to clarify the surface and shape (morphology) characteristics of the forms prepared. The SEM photographs show that the crystal particles of all the solvates were reduced to very small size (1–10 μm). The solvate-I and II showed irregular crystal structure and their surfaces were found to be uneven (Figure 4). The crystal shapes of all other solvates except solvate-VIII were not clearly defined and the appearance of both small and large particles is evident from the photomicrographs. Electron microscopy observations of solvate-VII showed more irregular crystals in habit but more uniform in size. The particles of solvate-VIII showed discrete, smooth, uniformly sized spherical appearance. The smooth surfaces and homogeneous morphology is further confirmed by the broadening of peaks in XRPD and ^13^C /MAS solid state NMR analysis.

### XRPD

**Figure 5:**
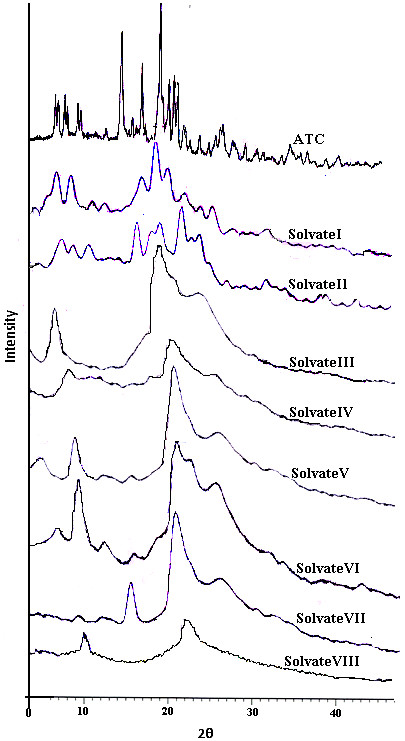
XRPD pattern of ATC and its solvates.

It is well known that XRPD is a powerful technique for the identification of crystalline and non-crystalline phases. This technique therefore was employed to establish differences in prepared solvates [11]. The XRPD pattern of ATC is characterized with a high degree of crystallinity as can be seen in Figure 5.

The XRPD pattern of solvate-I and II are readily distinguishable from all other solvates. Moreover, the position of peaks in the range 5-30 differentiate solvate-I that of solvate-II. The weak and broad nature of peaks indicated low crystalline nature of the forms. However, the XRPD spectra in all other solvates except solvate-VIII, showed two broad peaks of medium strength in the range 5.3±0.2 and 8.3±0.2 degree2θ and one large peak in the range 18-23 degree2θ with a maximum at about 18.3±0.2 degrees two-theta, instead of many peaks as appeared in solvate-I and II. The XRPD of solvate-VIII depicted one very small peak and another broad peak suggesting the very low crystalline nature of this form. The Figure 5 shows clear differences in the XRPD pattern of the solvates compared with the commercial sample.

**Figure 6:**
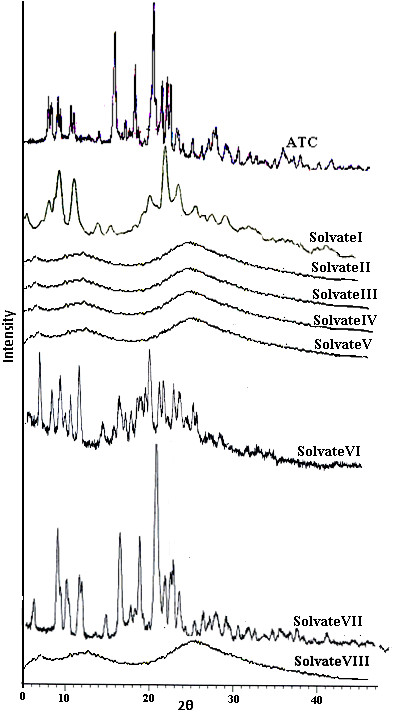
XRPD pattern of ATC and its solvates after desolvation.

These solvates were heated in a vacuum oven to generate desolvated forms and the XRPD pattern of these desolvated forms (Figure 6) showed that the solvates have converted to some new forms. The diffraction pattern of solvate-I was found to be similar after desolvation indicating that the crystal lattice has been retained when isopropyl alcohol leaves the lattice but the crystallinity increased up to some extent. The XRPD pattern of Solvate-II, III, IV, V and VIII indicated similar amorphous halo pattern after removal of the entrapped solvent. Based on this observation the solvates have been proposed to be different pseudopolymorphs having isomorphic structure. This similarity in XRPD patterns drug solvates having isomorphous nature of crystals has been reported in literature [1]. The Solvate-VI shows a different XRPD pattern and the new peaks observed are attributed to its conversion to a new crystalline form after desolvation. The XRPD pattern of solvate-VII after desolvation reveals that the pattern becomes same to that of the original sample indicating it has converted to drug after desolvation.

### ^13^C /MAS NMR analysis

**Figure 7:**
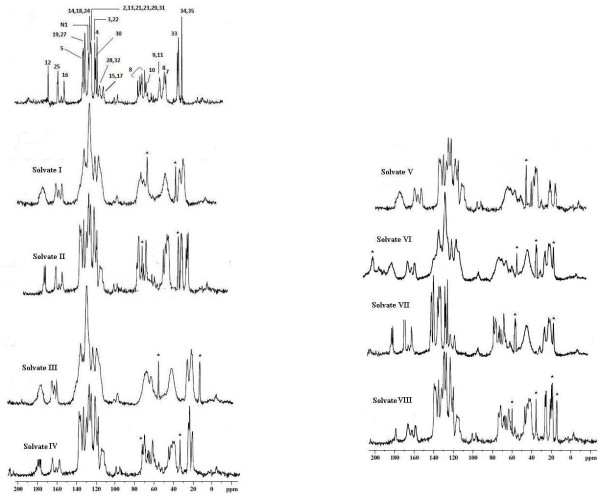
^**13**^**C /MAS solid state NMR spectra of ATC and its solvates (identified solvent resonances as star).**

The ^13^C /MAS solid state NMR spectra of ATC and its crystal forms recrystallised from various organic solvents were performed to futher confirm the presence of entrapped solvent in the crystal lattice of the solvate [17-20].The peak assignments are based on the comparison with the ^1^H and ^13^C NMR of dissolved atorvastatin (Figure 7). The resulting spectra of solvate I, solvate III and solvate VI of atorvastatin showed similar pattern, although noticeably distinct peaks for solvents were detected. In solvate I peaks at position 28.9 and 64.7 are associated with CH_3_ and CH group of isopropyl alcohol (IPL) indicating the presence of IPL in the solvate. The peaks at 16.9 and 58.9 in solvate III were most likely associated with ethanol solvent in crystal lattice. The common peaks at 16.9 and 58.9 were associated with a CH_3_ group of ethanol. In solvate VI peaks at 17.1 and 59.3 are also assigned to ethanol, while peaks at 34.1 and 203.4 were associated with acetone. The sharpness of peaks between 60–80 ppm which were assigned as C-8 and C-10 in solvate I,decreases in the crystal form of solvate III and solvate VI. However, in solvate II, solvate V the resolution of peaks was sharp. An extra peaks at 29.1 and 76.4 were detected in the solvate II which can be accounted for THF molecule. In solvate V an extra sharp signal at 49.8 was assigned to methanol. In solvate VII peaks at 16.1 and 58.3 are also assigned to ethanol. In solvate VIII an extra sharp peaks at 14.5, 18.1, 34.3 and 62.6 were due to butanol. It is revealed from ^13^C /MAS solid state NMR spectra of solvate VIII, the resolution of peak were sharp indicating it to be crystalline. It is interesting to note that the CH_3_ has resonances with different chemical shifts despite similar crystallographic packing. Thus, it is proposed that different molecular structures or further packing of crystal forms may be attributed to the residual solvent or solvate molecule within the crystal forms [20].

### Calorimetric studies

Microreaction-calorimetric studies were used to differentiate the solvates by determining their heat capacity as well as enthalpy of solutions.

### Heat capacity

The comparison of molar heat capacities of solvates with the drug gives information about the solvent molecule within the condensed phase solvates. The heat capacity of the solvation (ΔCp) is given by the equation 2 and is given in Table 2.

(2) $$\Delta {\text{CP}}_{\text{p}}\;=\;{\text{C}}_{\text{p}\left(\text{solvate}\right)}\;-\;\left({\text{C}}_{\text{p}\left(\text{drug}\right)}\;+\;{\text{xC}}_{\text{p}\left(\text{solvent}\right)}\right)$$

C_p(solvate)_ = molar heat capacity of solvate

C_p(drug)_ = molar heat capacity of atorvastatin calcium

C_p(solvent)_) = molar heat capacity of solvent in the liquid state (the value varies according to stoichiometry of solvates)

X = ratio of the stoichiometry of solvent to drug.

**Table 2:** **Molar heat capacities of the solvate formation ΔC**_**p**_**= (C**_**p(solvate)**_**- C**_**p(drug)**_**- C**_**p(solvent)**_**)**

	**Heat capacity at 25°C (J/g/K)**	**Molar heat capacity at 25°C (J/(mol.K)**	**Molar heat capacity of solvent (J/(mol.K) at 25°C**	**ΔC**_**p**_**at 25°C (J/(mol.K)**
**Atorvastatin (anhydrous) (ATC)**	1.25±0.014	1514.43±0.11	-	-
**Solvate- I (ATC + 0.5 Isopropyl alcohol)**	1.96±0.024	2347.86±0.05	77.0	756.42
**Solvate- II (ATC + THF)**	2.07±0.003	2547.11±0.04	123.0	909.68
**Solvate- III (ATC + Ethanol)**	1.98±0.007	2383.56±0.02	112.4	756.72
**Solvate- IV (ATC + 0.5 t-butanol)**	2.44±0.004	2913.66±0.12	56.2	1343.02
**Solvate- V (ATC + methanol)**	2.23±0.019	2720.96±0.12	159.0	1047.52
**Solvate-VI (ATC + 1 Acetone +0.5 Ethanol)**	2.43±0.007	3009.49±0.05	181.7	1313.35
**Solvate-VII (ATC + 0.5 Ethanol + Water)**	1.83±0.011	2234.91±0.06	131.4	589.10
**Solvate- VIII (ATC + 1- butanol)**	2.30±0.008	2840.19±0.02	174.3	1151.49

The comparison of molar heat capacities of solvates with the anhydrous drug gives information about the solvent molecule within the condensed phase solvates. The heat capacity was found to be higher for the solvated amorphate as compared to pure drug. The large ΔC_p_ for all the solvates suggest that they are present in the lattice channels (Table 2).

### Enthalpy of solution

As indicated by XRPD studies these forms are low crystalline in nature and the extent of crystallanility varies. The lattice energy was determined utilizing calorimetric studies which determine the enthalpy of solution which is closely associated with their lattice energy.

Molar enthalpy of solution (ΔH_sol_) was determined in phosphate buffer (pH8) as well as in methanol. All the solvates showed endothermic behavior in both phosphate buffer (pH8) and methanol (Table 3). The solvate-VIII was least endothermic (2.475 kJ mol^-1^) suggesting it to be most amorphous. The magnitude of molar enthalpy of solution of ATC is found to be highest (21.89 kJ mol^-1^) exhibiting minimum ease of molecular release from the lattice.

**Table 3:** Enthalpies of solution in phosphate buffer (pH8) and methanol at 25°C, solubility, enthalpies of solvation of Atorvastatin and its solvates

	**Solubility in phosphate buffer pH7 (mg/ml) (S**_**sol**_**pH7 )**	**Molar enthalpy of solution in its own solvent (kJ mol**^**-1**^**)**		**Enthalpy of solvation Δ**_**solv**_**H (kJ mol**^**−1**^**)**	**Molar enthalpy of solution (kJ mol**^**-1**^**)**	
		**Solvates (Δ**_**sol**_**H**^**2**^**)**	**Atorvastatin Calcium (ATC) (Δ**_**sol**_**H**^**1**^**)**		**Phosphate buffer (pH8) Δ**_**sol**_**H**	**Methanol Δ**_**sol**_**H (methanol)**
**Atorvastatin Calcium (ATC)**	0.166	−	−	−	21.89	19.11
**Solvate- I (ATC + 0.5 Isopropyl alcohol))**	0.374	−24.45	−13.52	−10.93	2.51	1.15
**Solvate- II (ATC + THF)**	0.192	−11.32	10.85	−22.17	14.31	9.95
**Solvate- III (ATC + Ethanol)**	0.292	−7.61	32.06	35.55	5.46	3.05
**Solvate- IV (ATC + 0.5 t-butanol)**	0.225	3.16	34.85	−31.69	11.50	5.45
**Solvate- V (ATC + methanol)**	0.341	2.12	16.35	−19.98	3.85	2.12
**Solvate-VI (ATC + 1 Acetone +0.5 Ethanol)**	0.245	−10.80	41.20	−48.40	8.35	5.1
**Solvate-VII (ATC + 0.5 Ethanol + Water)**	0.185	−4.25	112.49	−116.74	16.98	12.7
**Solvate- VIII (ATC + 1- butanol)**	0.387	−4.6	13.36	−17.96	1.17	0.57

As discussed above, the endothermic ΔH_sol_ in buffer and ΔH_sol_ in methanol follows the order: solvate-VIII (1-butanol solvate) < solvate-I (isoproplyate) < solvate-V (methanol solvate) < solvate-III (ethonalate) < solvate-VI (acetone ethanol solvate) < solvate-IV (t-butanol solvate) < solvate-II (THF solvate) < solvate-VII (mixed hemi-ethanol hydrate) < ATC.

**Figure 8:**
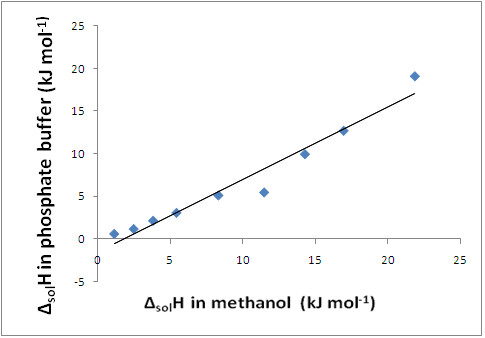
Plot between enthalpy of solution of ATC solvates in phosphate buffer pH8 and in Methanol.

The enthalpies of solution for all the solvates is found to be in the same order in both the solvents as indicated by a straight line observed in the plot between the enthalpies of solution in the two solvents confirming perfect correlation between two sets of data (Figure 8).

In order to determine the enthalpy associated with the solvation process the enthalpy of solution of a particular solvate was determined in its own solvent. The enthalpy of solvation [21] was calculated by the equation (3) and the values are given in Table 3.

(3) $$\Delta_{\text{soly}}\text{H}=\Delta_{\text{sol}}{\text{H}}^{1}-\Delta_{\text{sol}}{\text{H}}^{2}$$

Δ_sol_H^2^ = enthalpy of solution of the drug

Δ_sol_H^1^= enthalpy of solution of the solvate in solvent of solvation

**Figure 9:**
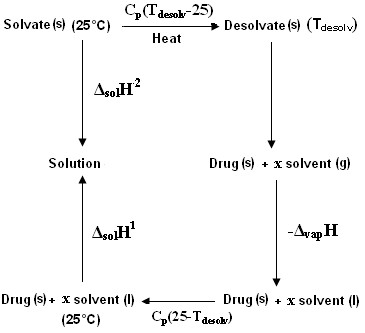
**Thermodynamic cycle.** (Where, x = ratio of the stoichiometry of solvent to drug).

If we now consider the following thermodynamic cycle (Figure 9).

The enthalpies of solution of the drug and the solvates, the enthalpy of vapourization of the solvent and the heat capacities of the drug, solvate and the solvent and further assuming that heat capacities remain constant during the temperature range 25°C to T_desolv_ the enthalpy of desolvation is given by the equation:

(4) $$\begin{array}{l} \Delta_{\text{desoly}}\;\text{H}\;=\;\Delta_{\text{soln}}{\text{H}}^{2}\;-\;\Delta_{\text{soln}}{\text{H}}^{1}\;-\;\Delta \text{CP}\left({\text{T}}_{\text{desoly}}\;-\;25\right)\\ \;\;+1/2\;\Delta_{\text{yap}}\text{H} \end{array}$$

The measured values of enthalpy of desolvation from DSC and those calculated from equation (3) are compared in Table 4. The agreement seems to be reasonable in spite of several assumptions and the measured heat capacity for the solvates may be C_s_ and not C_p_.

**Table 4:** **The enthalpy of desolvation (T**_**desolv**_**) of different solvates of ATC**

	**Enthalpy of desolvation, Δ**_**desolv**_**H (kJ mol**^**−1**^**) from DSC**	**Enthalpy of desolvation, Δ**_**desolv**_**H (kJ mol**^**−1**^**) calculated from equation 4**
**Atorvastatin (ATC)**	-	
**Solvate- I (ATC + 0.5 Isopropyl alcohol)**	24.20	25.93
**Solvate- II (ATC + THF)**	51.47	57.17
**Solvate- III (ATC + Ethanol)**	44.69	34.39
**Solvate- IV (ATC + 0.5 t-butanol)**	78.85	78.31
**Solvate- V (ATC + methanol)**	26.27	26.70
**Solvate-VI (ATC + 1 Acetone +0.5 Ethanol)**	56.74	52.01
**Solvate-VII (ATC + 0.5 Ethanol + Water)**	104.64	103.96
**Solvate- VIII (ATC + 1- butanol)**	28.56	31.81

### Solubility Studies

The solubility studies of pure drug and various solvates were performed in water and phosphate buffer pH 6.8 at 37°C and are given in table 3. The solubility study suggests that the most amorphous form (solvate-VIII) was most soluble (0.374±0.06 mg/ml) with a 2.07 times increase in solubility in comparison to commercial sample while the commercial sample which is crystalline was least soluble.

### Effect of ATC & various ATC solvates on lipid profile

#### In vivo studies in rats

**Figure 10:**
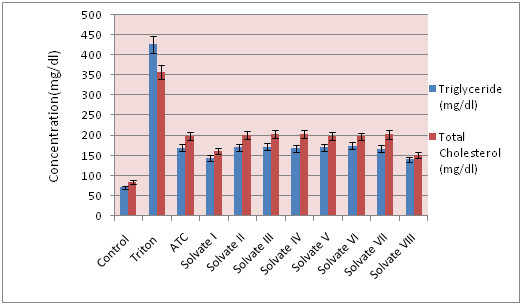
Graph representation of effect of ATC & various solvates on lipid profile Concentration (mg/dl).

Hypolipidemic drugs like ATC (HMG-CoA reductase inhibitors) are known to reduce elevated total cholesterol and TG levels in blood. At the same time they cause elevation of the HDL-cholesterol levels, which promote the removal of cholesterol from peripheral cells and facilitate its delivery back to the liver. This pharmacodynamic effect is reported to be dose dependent hence, it was used as a basis for the comparison of in vivo performance of ATC and its solvates. Administration of Triton significantly increased triglyceride and total cholesterol levels in rats and leads to hypercholesterolemia. The test group showed significant decrease in total cholesterol and in TG. Thus, solvate-I and solvate-VIII performed better than pure ATC in reducing total cholesterol and TG levels (Table 5 & Figure 10). This could be primarily attributed to the improved solubility associated with different forms between ATC and its various solvates.

**Table 5:** Effect of ATC & various ATC solvates on lipid profile

**Groups**	**Triglyceride (mg/dl)**	**Total Cholesterol (mg/dl)**
**I Control**	71.3	84
**II Triton**	426	357
**III ATC**	**169**	**198**
**IV Solvate I**	**143**	**161**
**V Solvate II**	170	201
**VI Solvate III**	172	203
**VII Solvate IV**	168	204
**VIII Solvate V**	170	198
**IX Solvate VI**	174	197
**X Solvate VII**	167	202
**XI Solvate VIII**	**140**	**152**

Triton significantly increased triglyceride and total cholesterol levels in rats. ATC reduced lipid profile in triton treated rats. However, solvate-VIII and solvate-I showed better lipid lowering profile as compared to atorvastatin against triton-induced dyslipidemia.

## Experimental

Recrystallization of the drug was carried out by dissolving ATC in minimum amount of solvent mixtures at temperature few degrees below the boiling point of the solvent. The obtained suspension was stirred at ambient temperature for a sufficient time (24–48 hrs). The saturated solution was then filtered while hot (0.45 μm millipore microfilter) and were left to recrystallize by spontaneous cooling and evaporation at room temperature. Recrystallized product obtained in a period of 15–21 days was isolated by suction filtration and dried at ambient temperature under vacuum prior to solid and liquid state analysis. Based upon the solvents used for recrystalization, the solvates were designated as solvate-I (hemi isopropylate), solvate-II (THF solvate), solvate-III (ethanol solvate), solvate-IV (t-butanol solvate), solvate-V (methanol solvate), solvate-VI (acetone ethanol solvate), solvate-VII (hemi-ethanol hydrate) and solvate-VIII (1- butanol solvate). These solvates were heated in a vacuum oven to generate desolvated forms. Solvate I, Solvate II and Solvate VII was heated upto 120°C; Solvate III, Solvate IV, Solvate V, Solvate VI was heated upto 110°C and Solvate VIII was heated up to 130°C. The each form was heated in a vacuum oven for 4 hours.

### Thermal analysis

DSC thermograms were obtained on DSC, Q20, TA Instruments-Waters LLC, USA. The calorimeter was calibrated for temperature and heat flow accuracy using the melting of pure indium (mp 156.6°C and ΔH of 25.45 Jg^−1^). A mass between 2–8 mg was taken into the aluminium pan, covered with lid and sealed. DSC curves were obtained under a nitrogen purge of 50 mL per minute at a heating rate of 10°C per minute with the temperature range from 50-350°C.

Loss of solvent from the samples was characterized by TGA obtained by TA Instruments-Waters LLC, USA. TGA traces were recorded at heating rates of 10°C per minute under a nitrogen purge of 50 ml per minute. Samples with masses between 1–10 mg were analyzed using platinum pan. Mass loss (%) was calculated based on the mass of the original sample.

### Gas chromatography–mass spectrometry (GC-MS) Analysis

GC-MS analysis was carried out on a GC Clarus 600 Perkin Elmer system equipped with head space turbo matrix-16 and AOC-20i autosampler. Gas chromatograph interfaced to a mass spectrometer (GC-MS) instrument employing the following conditions: Column Elite-1 fused silica capillary column (30 mm×0.25 mm ID ×1 μM df, composed of 100% Dimethyl poly siloxane), operating in electron impact mode at 70 eV; helium (99.999%) was used as carrier gas at a constant flow of 1ml/min and an injection volume of 0 μl was employed (split ratio of 50:1) injector temperature 250°C; ion-source temperature 280°C. The oven temperature was programmed from 35°C (isothermal for 8 min), with an increase of 10°C/min, to 150°C, hold 2 min then 5°C/min to 200°C. Mass spectra were taken at 70 eV; a scan interval of 0.5 seconds and fragments from 30 to 200 Da.

### Nuclear Magnetic Resonance (NMR) Spectroscopy

NMR spectra were obtained in deuterated D_2_O on Bruker Advance II 400 NMR spectrometer operating at 400 Hz.

### X-Ray powder diffraction Analysis

The powder diffraction patterns were recorded on an X-ray diffractometer (XPERT-PRO, PAN analytical, Netherlands, Holand) with Cu as tube anode. The diffractograms were recorded under following conditions: voltage 40 kV, 35 mA, angular range 5 and fixed divergence slit change. Care was taken to avoid crystal changes during sample preparation. Approximately 200 mg of samples were loaded into the sample holder, taking care not to introduce preferred orientation of the crystals.

### Solid State Nuclear Magnetic Resonance spectroscopy (SSNMR)

The solid state ^13^C cross-polarization magic angle spinning (CP-MAS) spectra were acquired using a Bruker DSX-300 NMR spectrometer using a 5 mm double resonance CP-MAS probe. The ^13^C resonance frequency is 75.46 MHz and the data was collected at 273 K using a spectral width of 18.8 kHz, 1130 complex data points, acquisition time 30 ms, contact time 2 ms. A total of 1024 scans were acquired with a relaxation delay 4 s. All the spectra were referenced to tetramethylsilane (TMS) using the carbonyl carbon of glycine (176.03 ppm) as a secondary reference.

### Calorimetric study

#### Heat capacity measurement

Heat capacity measurements were performed on Microreaction calorimeter obtained from Thermal Hazards Technology, UK. The size of sample used in analysis ranged from 100-150 mg and was weighed (Sartorius Model CP225D) into a glass vial. The empty vial was placed as the reference sample. A temperature step of 2 C is then applied to the system and the heat required is measured. The experiment is then repeated in the reverse direction to verify the measurement. A measurement with empty vial was conducted first to ensure any difference between the heat capacity of the vials is accounted for. The heat capacity for ATC and all the solvates was conducted at 25°C (24-26°C).

#### Enthalpy of solution measurements

Calorimetric studies were performed on Microreaction calorimeter obtained from Thermal Hazards Technology, UK. Two different solvents, viz, the phosphate buffer (pH 2) and methanol were used to determine the enthalpy of solution and the measurements were performed at 25°C. The size of sample used in this study ranged from 10 to 20 mg and was weighed (Sartorius Model CP225D) into a cylindrical glass tube covered with parafilm on one side. This cylindrical tube was submerged into the ampoule containing the solvent. A plunger with a cap was put from the open end of the tube. The same solvent was put into the reference ampoule. These were put into the sample and reference holes until both rest on the base of the holes. The apparatus was maintained at constant temperature at 25°C (±0.0005°C). The parafilm was shattered mechanically by means of plunger.

#### Aqueous solubility measurement

MSW-275 (Macro scientific works, New Delhi) shaker was used for measuring aqueous solubility of solvates of ATC. Solubility studies were performed by adding 10 mg of ATC or its solvates in flasks containing 10 mL of phosphate buffer pH 6.8. The mixtures were shaken at 37°C for 24 h. The aliquots were filtered through 0.45 μm membrane filter and analyzed spectrophotometrically at 242 nm. The standard plot of ATC was prepared by dissolving a weighed amount of the drugs in phosphate buffer pH 6.8, suitably diluted and absorbances taken at wavelength 242 nm on a spectrophotometer. E^1%^_1cm_ was calculated.

## Conclusion

The study provides insights into the thermal characterization of ATC and its various forms. The characterization of crystalline solvated forms of ATC has suggested the formation of eight solvates by entrapment of the solvent molecules in the cavities formed by the host drug. The amounts of organic solvents in these forms as drug substances were below the limit regulated by the ICH guideline Q3C on residual solvents in pharmaceuticals [22]. The GC-MS analysis established the solvent associated with ATC as mixtures of solvents have been used for recrystallization of the solvates. The solvent molecules incorporated into the drug molecule were detected by ^13^CP/MAS solid state NMR spectroscopy. ^13^C Solid state NMR provides an excellent advantage over other techniques in its ability to distinguish different solvates of very similar crystallographic structure. All the forms were endothermic in phosphate buffer pH8. The solvate-VIII was found to most soluble among all the solvates. The hypolipidemic activity study indicated solvate I and VIII to be most effective in reducing the cholesterol and triglyceride levels. This could be primarily attributed to the improved solubility associated with these solvates in comparison to ATC and its various solvates.

## Methods

### Drugs and reagents

Triton was purchased from Sigma (St. Louis, MO, USA). Atorvastatin was obtained as gift sample from Ind-Swift Lab, Mohali. Triglyceride and total cholesterol kits were procured from Span Diagnostics.

### In vivo studies in rats

#### Animals

The hypolipidemic activity of ATC and its different solvates was determined in male Wistar rats (220–260 g) bred in Central Animal House facility of Panjab University. The rats were housed under optimal laboratory conditions, maintained on 12 h light and dark cycle and had free access to food (Hindustan Lever Products, Kolkata, India) and water. Animals were acclimatized to laboratory conditions before the tests. All experiments were carried out between 0900 and 1700 h. The experimental protocols were approved by the Institutional Animal Ethics Committee and conducted according to the Indian National Science Academy guidelines for the use and care of animals.

#### Induction and assessment of cholesterol

Fifty five male Wistar rats were divided into the following 11 groups containing 5 animals in each group:

Group I. Control (Normal saline)

Group II. Triton (200 mg/kg, i.p.) induced hyperlipidemia

Group III. Triton treated rats received ATC (30 mg/kg, oral gavage)

Group IV. Triton treated rats received ATC S1 (30 mg/kg, oral gavage)

Group V. Triton treated rats received ATC-S2 (30 mg/kg, oral gavage)

Group VI. Triton treated rats received ATC-S3 (30 mg/kg, oral gavage)

Group VII. Triton treated rats received ATC-S4 (30 mg/kg, oral gavage)

Group VIII. Triton treated rats received ATC-S5 (30 mg/kg, oral gavage)

Group IX. Triton treated rats received ATC-S6 (30 mg/kg, oral gavage)

Group X. Triton treated rats received ATC-S7 (30 mg/kg, oral gavage)

Group XI. Triton treated rats received ATC-S8 (30 mg/kg, oral gavage)

Atrovastain and various solvates were freshly suspended in 0.5% carboxymethyl cellulose and administered by oral gavage. Two hours later, all groups except control animals received intraperitonial injection of Triton (100 mg/kg). Eighteen hours after triton injection, blood was collected from tail vein. Plasma was separated and stored at −20°C for further biochemical analysis. Total cholesterol and triglyceride were analyzed in plasma samples using diagnostic kits as per manufacturer instructions.

## Competing interests

The authors declared that they have no competing interest.

## Authors’ contributions

AK prepared the samples and carried out all the experiments. AK drafted the manuscript, and all authors read and approved the final manuscript.

## Supplementary Material

Additional file 1 **Figure S1. **^1^H NMR of ATC (liquid state) and its solvates. Click here for file
