# Supplementary figures and images for: Characterisation and evaluation of pharmaceutical solvates of Atorvastatin calcium by thermoanalytical and spectroscopic studies
Source: Chem Cent J. 2012 Oct 6;6:114. doi: 10.1186/1752-153X-6-114 (PMC3547732; doi:10.1186/1752-153X-6-114)

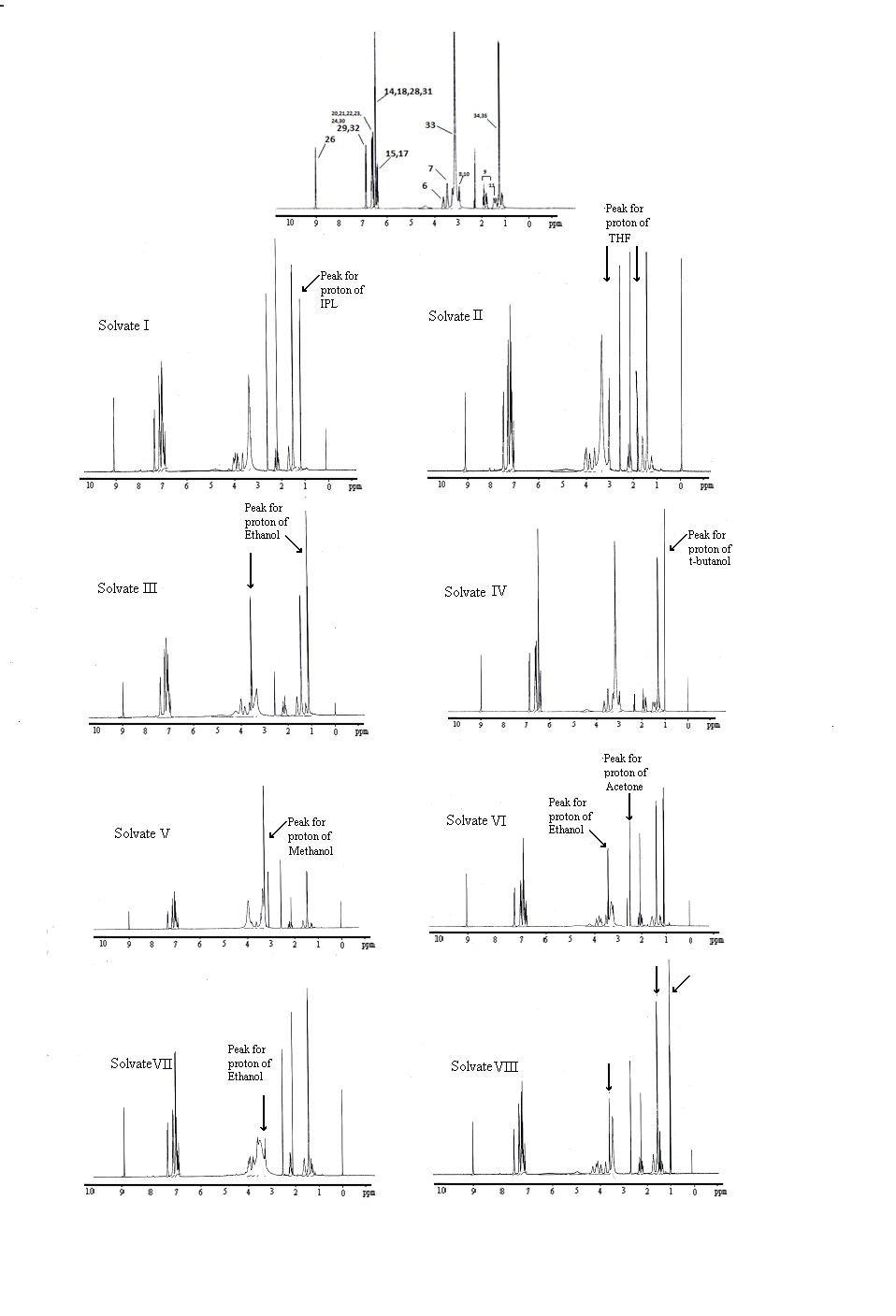

Supplement: Additional file 1 — Figure S1. 1H NMR of ATC (liquid state) and its solvates. [file 1752-153X-6-114-S1.tiff]
